# Supplementary material for: Sera Protein Signatures of Endometrial Cancer Lymph Node Metastases
Source: Int J Mol Sci. 2022 Mar 18;23(6):3277. doi: 10.3390/ijms23063277 (PMC8954239; doi:10.3390/ijms23063277)
Supplement: Supplementary file 1 [file ijms-23-03277-s001.zip › Supplementary.pdf]

**Supplementary A: Supplementary Information Word Files. File A1**: Author comments concerning the Ovarian data previously published; **File A2**: Patient samples utilized for MSMS; **File A3**: Binary LOOCV test metrics; **File A4**: Concerning Files uploaded to JPost repository; **File A5**: LCQ-ADVANTAGE Instrument Method for MSMS example; **File A6**: LCQ-ADVANTAGE Machine Status Log MSMS example).

**Supplementary B: Supplementary Information Excel Files. File B1**: Sera as used in LOOCV Figures; **File B2**: Master peak areas for each patient De-identified; **File B3**: LOOCV Significant peaks by test and figure; **File B4**: LOOCV results of sera MS peaks; **File B5**: Peak list for MSMS analysis of sera).

Figure S1. LOOCV Analysis of Randomized Groups.
Figure S2. LOOCV Analysis of Stages I or III Ovarian and Endometrial Cancers.
Table S1. Gene IDs and Names of proteins.
Supplementary A: Supplementary Information Word Files. File A1: Author comments concerning the Ovarian data previously published; File A2: Patient samples utilized for MSMS; File A3: Binary LOOCV test metrics; File A4: Concerning Files uploaded to JPost repository; File A5: LCQ-ADVANTAGE Instrument Method for MSMS example; File A6: LCQ-ADVANTAGE Machine Status Log MSMS example).

Supplementary B: Supplementary Information Excel Files. File B1: Sera as used in LOOCV Figures; File B2: Master peak areas for each patient De-identified; File B3: LOOCV Significant peaks by test and figure; File B4: LOOCV results of sera MS peaks; File B5: Peak list for MSMS analysis of sera).
